# Supplementary material for: Aroma Compounds in Essential Oils: Analyzing Chemical Composition Using Two-Dimensional Gas Chromatography–High Resolution Time-of-Flight Mass Spectrometry Combined with Chemometrics
Source: Plants (Basel). 2023 Jun 18;12(12):2362. doi: 10.3390/plants12122362 (PMC10305201; doi:10.3390/plants12122362)
Supplement: Supplementary file 1 [file plants-12-02362-s001.zip › plants-2425759-supplementary.pdf]

Aroma Compounds in Essential Oils: Analyzing Chemical Composition using Two-Dimensional Gas Chromatography-High Resolution Time-of-Flight Mass Spectrometry Combined with Chemometrics

Nemanja Koljančić, Olga Vyviurska, Ivan Špánik *

Institute of Analytical Chemistry, Faculty of Chemical and Food Technology, Slovak University of Technology in Bratislava, Radlinského 9, 812 37 Bratislava, Slovakia

---

Corresponding author

*e-mail: ivan.spanik@stuba.sk Tel.: +421-2-59325277

Table S1 100 features identified with MEGA-DEX DET stationary phase in ^{1}D as true positives by tile-based F-ratio analysis, along with retention information

Feature no.	Name	Similarity	Probability	CAS	Quant mass	*F*-ratio	Mean $^{1}t_R$ (sec)	Mean $^{2}t_R$(sec)	%RSD $^{1}t_R$	%RSD $^{2}t_R$
1	Geraniol	918	6674	106-24-1	69	2.64E+15	2056	3.36	0	0
2	Phenethyl alcohol	940	9506	60-12-8	91	5.57E+14	1736	3.23	0	4.27E-14
3	Nerol	940	7760	106-25-2	69	5.12E+14	1992	3.14	0	0
4	Farnesol	929	3256	3790-71-4	69	3.98E+14	4056	4.11	0	0
5	Citronellol I	930	5264	106-22-9	69	3.81E+14	1912	3.32	0	0
6	Phenylethyl tiglate	946	9502	55719-85-2	104	1.90E+14	3392	6.06	0	3.03E-14
7	β-Eudesmol I	896	6331	473-15-4	59	1.72E+14	3624	5.26	0	0
8	Citronellyl formate	956	6287	105-85-1	69	1.65E+14	1840	3.76	0	3.67E-14
9	epi-γ-Eudesmol	919	5835	1209-71-8	189	1.61E+14	3448	5.32	0	3.46E-14
10	β-Myrcene	852	2616	123-35-3	69	1.56E+14	2008	4.15	0	2.22E-14
11	Geranial	914	8798	141-27-5	69	1.50E+14	1912	4.69	0	1.96E-14
12	Isomenthone I	951	5497	491-07-6	112	1.06E+14	1352	3.77	0	3.66E-14
13	Citronellol II	850	3500	106-22-9	81	1.05E+14	1976	2.95	0	1.56E-14
14	Geranyl acetate	889	3238	141-12-8	69	9.83E+13	2376	4.46	0	0
15	*n*-Hexadecanoic acid	850	5332	57-10-3	57	9.79E+13	5192	3.23	0	4.27E-14
16	Geranyl tiglate	894	7374	7785-33-3	69	9.72E+13	3776	5.00	0	0
17	γ-Eudesmol	917	5857	1209-71-8	189	9.29E+13	3536	4.92	0	1.87E-14
18	Linalool	944	8299	78-70-6	71	8.53E+13	1224	2.77	0	3.32E-14
19	Isomenthone II	946	5312	491-07-6	112	7.89E+13	1448	3.88	0	2.37E-14
20	Calamenene	817	9142	73209-42-4	159	7.82E+13	2936	5.00	0	0
21	Methyl geranate	881	7536	2349-14-6	69	7.72E+13	2104	4.32	0	2.13E-14
22	Citronellyl butyrate	968	6355	141-16-2	81	7.47E+13	2944	4.15	0	2.22E-14
23	Geranic acid	964	7856	4698-08-2	69	7.20E+13	2960	2.93	0	0
24	Phenethyl acetate	933	8575	103-45-7	104	5.78E+13	1912	5.20	0	1.77E-14
25	Terpinen-4-ol	925	6976	562-74-3	71	5.42E+13	1552	3.48	0	3.96E-14
26	Neryl propionate	902	3165	105-91-9	69	4.49E+13	2768	4.50	0	0
27	Eugenol	950	6575	97-53-0	164	3.94E+13	2488	4.97	0	0
28	α-Terpineol I	939	8177	98-55-5	59	3.24E+13	1672	3.55	0	2.59E-14

29	δ-Cadinene	941	6240	16729-01-4	161	3.06E+13	2920	4.44	0	2.07E-14
30	Myrcene	943	7664	123-35-3	93	2.94E+13	720	2.01	0	4.57E-14
31	α-Terpineol II	929	7965	98-55-5	59	2.41E+13	1696	3.47	0	2.65E-14
32	α-Pinene	937	6100	80-56-8	93	2.33E+13	624	5.97	0	0
33	Geranyl isobutyrate	873	2395	105-91-9	69	2.09E+13	3136	4.50	0	0
34	Methyleugenol	935	8209	93-15-2	178	1.96E+13	2608	5.97	0	0
35	Rose oxide I	884	9543	16409-43-1	139	1.93E+13	1152	3.08	0	2.98E-14
36	Rose oxide II	893	9230	16409-43-1	139	1.88E+13	1184	2.95	0	1.56E-14
37	Rose oxide III	879	9519	16409-43-1	69	1.45E+13	1256	3.11	0	0
38	Ledene	912	2770	21747-46-6	105	1.39E+13	2792	4.27	0	4.31E-14
39	2-Phenylethyl 2-methylbutanoate	962	7808	24817-51-4	104	1.34E+13	2880	5.30	0	1.73E-14
40	*n*-Hexanol	879	7483	111-27-3	56	1.32E+13	664	1.53	0	1.50E-14
41	Hexadecanal	938	2517	629-80-1	55	1.28E+13	4160	4.22	0	0
42	Citronellyl tiglate	938	6744	24717-85-9	81	1.28E+13	3576	4.56	0	2.02E-14
43	α-Calacorene	944	8997	21391-99-1	157	1.22E+13	3056	5.26	0	0
44	Caryophyllene	963	5634	13877-93-5	93	1.2E+13	2424	4.24	0	2.17E-14
45	Germacrene D	930	6150	23986-74-5	161	1.15E+13	2728	4.34	0	4.24E-14
46	Neryl butyrate	891	4900	2345-26-8	93	1.12E+13	2904	4.35	0	2.11E-14
47	β-Bisabolene	845	1151	495-61-4	69	1.02E+13	3960	4.00	0	0
48	Citronellyl isohexanoate	927	3353	71662-18-5	81	9.28E+12	3600	4.17	0	2.20E-14
49	Aristolene	893	1037	134-28-1	105	8.89E+12	3568	5.06	0	1.82E-14
50	Levandulyl acetate	943	5577	25905-14-0	69	8.51E+12	1880	4.00	0	0
51	Citronellyl propionate	952	5959	141-14-0	69	8.33E+12	2568	4.11	0	0
52	α-Linolenic acid	846	2617	463-40-1	55	7.49E+12	5808	4.35	0	2.11E-14
53	Geranyl propionate	845	3239	105-90-8	69	7.41E+12	3288	4.39	0	0
54	β-Bourbonene II	916	9557	5208-59-3	81	7.38E+12	2232	3.83	0	3.60E-14
55	Farnesal	911	8744	502-67-0	69	6.41E+12	3944	5.27	0	3.49E-14
56	Neryl hexanoate	888	2047	68310-59-8	69	6.41E+12	3792	4.50	0	0
57	Widdrol hydroxyether	745	4393	29620-91-5	43	5.96E+12	3656	5.28	0	0
58	Junenol	927	5075	472-07-1	109	5.94E+12	3384	5.07	0	3.63E-14
59	α-Farnesene	951	6798	502-61-4	93	5.72E+12	2840	4.02	0	4.57E-14
60	β-Dihydroagarafuran	881	8603	5956-09-2	137	5.71E+12	2808	4.77	0	3.85E-14

61	Nonanal	928	8286	124-19-6	57	5.55E+12	1128	3.12	0	1.47E-14
62	β-Bourbonene III	914	8727	119903-95-6	81	5.37E+12	3048	4.59	0	4.01E-14
63	Epicubenol	926	5038	19912-67-5	161	5.29E+12	3400	4.84	0	3.80E-14
64	α-Humulene	919	7126	6753-98-6	93	5.07E+12	2592	4.26	0	2.16E-14
65	Guaiol	904	4582	220766-71-2	107	4.84E+12	3328	4.72	0	0
66	β-Eudesmol II	860	4837	473-15-4	105	4.60E+12	3632	5.04	0	1.82E-14
67	Benzyl Benzoate	939	9813	120-51-4	105	4.29E+12	4184	7.48	0	3.69E-14
68	α-Agarofuran	964	9086	5956-12-7	82	4.17E+12	3072	5.30	0	1.73E-14
69	Geranyl hexanoate	899	3980	68310-59-8	69	3.71E+12	3944	4.59	0	4.01E-14
70	Spathulenol I	849	1377	72203-24-8	93	3.64E+12	2888	4.75	0	0
71	Phenethyl benzoate	959	9132	94-47-3	104	3.61E+12	4536	7.44	0	2.47E-14
72	α-epi-Cadinol	950	5593	19912-62-0	95	3.28E+12	3592	4.86	0	0
73	δ-Guaiene	932	5570	3691-11-0	93	3.27E+12	2824	4.32	0	2.13E-14
74	Tetradecyl octanoate	867	5197	16456-36-3	55	2.97E+12	6152	5.86	0	0
75	2-Phenylethyl geranoate	900	9433	/	105	2.71E+12	5256	6.48	0	4.26E-14
76	α-Guaiene	947	5067	3691-12-1	105	2.69E+12	2464	4.00	0	0
77	Squalene	836	4038	111-02-4	81	2.67E+12	4968	4.94	0	1.86E-14
78	Citronellyl valerate	910	5987	7540-53-6	81	2.66E+12	3360	4.19	0	2.19E-14
79	Guaia-6,9-diene I	952	3079	37839-64-8	105	2.49E+12	2504	4.15	0	2.22E-14
80	Neral	925	7261	106-26-3	41	2.43E+12	1792	4.43	0	2.08E-14
81	Neryl acetate	871	3545	141-12-8	69	2.32E+12	2256	4.33	0	2.12E-14
82	γ-Muurolene	937	4290	30021-74-0	161	2.32E+12	2672	4.35	0	2.11E-14
83	Phenethyl propionate	954	8324	122-70-3	104	2.22E+12	2328	5.31	0	1.73E-14
84	Nerolidol	940	4073	40716-66-3	69	1.90E+12	3200	4.06	0	2.26E-14
85	Dihydromyrcenol	897	8402	18479-58-8	59	1.86E+12	2840	3.12	0	1.47E-14
86	Guaia-6,9-diene II	903	1048	36577-33-0	91	1.63E+12	2528	4.27	0	4.31E-14
87	Drim-8(12)-ene II	841	5810	168113-16-4	95	1.60E+12	5768	5.70	0	3.23E-14
88	Caryophyllene oxide	901	5746	1139-30-6	79	1.50E+12	3232	5.31	0	1.73E-14
89	γ-Cadiene	926	3202	39029-41-9	161	1.39E+12	2856	4.56	0	2.02E-14
90	Norbourbonone	921	9050	13844-03-6	81	1.37E+12	3184	5.70	0	3.23E-14
91	α-Copaen-11-ol	870	728	25246-27-9	59	1.36E+12	3704	5.37	0	0
92	β-Farnesene	930	6687	18794-84-8	69	1.35E+12	2584	3.82	0	0

93	β-Bourbonene I	888	9509	119903-95-6	80	1.20E+12	2200	3.60	0	2.55E-14
94	Aromandendrene	916	2211	489-39-4	91	1.17E+12	2616	4.25	0	0
95	Spathulenol II	806	3438	77171-55-2	105	1.13E+12	3248	5.01	0	0
96	Globulol	932	3693	489-41-8	69	1.08E+12	3304	4.69	0	1.96E-14
97	Farnesyl acetone	833	2884	1117-52-8	69	1.07E+12	4608	4.96	0	1.85E-14
98	Drim-8(12)-ene I	848	5647	168113-16-4	95	1.07E+12	5160	5.58	0	1.65E-14
99	Phenylacetaldehyde	923	8272	122-78-1	91	9.49E+11	1096	3.95	0	2.33E-14
100	para-Cymene	924	6804	99-87-6	119	7.81E+11	856	2.53	0	1.82E-14

Table S2 100 features identified with Rt-βDEXsp stationary phase in ^{1}D as true positives by tile-based F-ratio analysis, along with retention information

Feature no.	Name	Similarity	Probability	CAS	Quant mass	*F*-ratio	Mean ^1t_R (sec)	Mean ^2t_R(sec)	%RSD ^1t_R	%RSD ^2t_R
1	Phenylethyl tiglate	939	9315	55719-85-2	104	4.10E+14	4026	3.69	0	2.49E-14
2	Citronellyl formate II	957	6814	105-85-1	69	2.51E+14	2436	2.46	0	1.87E-14
3	Menthone	941	5088	10458-14-7	112	2.37E+14	1980	2.67	0	3.44E-14
4	Geranyl formate	894	3786	105-86-2	69	2.19E+14	2586	2.69	0	0
5	Linalool I	927	8248	78-70-6	71	1.04E+14	1764	2.00	0	0
6	Linalool II	947	8768	78-70-6	71	9.58E+13	1806	1.96	0	3.52E-14
7	epi-γ-Eudesmol	900	5773	1209-71-8	189	9.26E+13	4026	3.43	0	0
8	Phenethyl alcohol	939	9603	60-12-8	91	8.99E+13	2298	2.27	0	0
9	Farnesol	923	4184	4602-84-0	69	8.15E+13	4614	2.85	0	3.23E-14
10	Linalyl acetate	915	6168	115-95-7	93	7.13E+13	2244	2.52	0	1.82E-14
11	Geranyl tiglate	916	8321	7785-33-3	69	7.08E+13	4350	3.25	0	0
12	Germacrene D	927	5631	23986-74-5	161	7.08E+13	3186	3.18	0	0
13	Geraniol	944	6910	106-24-1	69	6.11E+13	2628	2.51	0	3.66E-14
14	β-Eudesmol I	909	6962	473-15-4	59	5.90E+13	4272	3.43	0	0
15	Citronellyl formate I	875	4621	105-85-1	69	5.13E+13	2424	2.70	0	1.70E-14
16	Calamenene	805	9160	73209-42-4	159	5.09E+13	3438	3.45	0	2.66E-14
17	Citronellol	883	4585	106-22-9	81	4.11E+13	2508	2.18	0	0
18	Geranyl acetate	896	3372	141-12-8	69	4.03E+13	2928	2.92	0	3.15E-14

19	3,7-Dimethyl-1,7-octanediol	908	6756	107-74-4	59	3.99E+13	3456	2.16	0	2.13E-14
20	β-Phenylethyl butyrate	957	6877	103-52-6	104	3.88E+13	3096	3.20	0	1.44E-14
21	β-Eudesmol II	909	8882	473-15-4	59	3.69E+13	4284	3.32	0	0
22	Geranyl propanoate	912	3017	105-90-8	69	3.54E+13	3318	2.96	0	1.55E-14
23	Viridiflorene	921	2465	74409-93-1	105	2.80E+13	3204	3.17	0	2.90E-14
24	Geraniol butyrate	898	3961	106-29-6	69	2.74E+13	3702	3.06	0	1.50E-14
25	α-Terpineol	942	8402	98-55-5	59	2.74E+13	2328	2.29	0	2.01E-14
26	Nerol	860	6793	106-25-2	69	2.28E+13	2472	2.32	0	0
27	Valerianol	933	5748	20489-45-6	161	2.26E+13	4254	3.29	0	1.40E-14
28	δ-Cadinene	940	5679	16729-01-4	161	1.87E+13	3360	3.26	0	4.23E-14
29	α-Muurolene	914	961	10208-80-7	105	1.80E+13	3228	3.22	0	2.86E-14
30	Methyleugenol	922	8223	93-15-2	178	1.78E+13	3240	3.48	0	3.96E-14
31	Geranial	904	9014	141-27-5	69	1.74E+13	2658	2.61	0	0
32	Phenethyl propionate	949	5588	122-70-3	104	1.66E+13	2946	3.24	0	4.26E-14
33	Citronellyl tiglate	938	7369	24717-85-9	95	1.58E+13	4176	2.95	0	1.56E-14
34	Neryl hexanoate	899	2951	68310-59-8	69	1.27E+13	4362	2.98	0	3.09E-14
35	Aristolene	851	1398	6831-16-9	105	1.11E+13	4224	3.23	0	4.27E-14
36	Citronellyl propionate	942	5627	141-14-0	81	1.07E+13	3144	2.67	0	3.44E-14
37	2-Phenylethyl 2-methylbutanoate	970	8213	24817-51-4	104	1.07E+13	3504	3.26	0	4.23E-14
38	Rose oxide I	900	9554	16409-43-1	139	1.03E+13	1596	2.28	0	2.02E-14
39	1,2-Hexadecene epoxide	873	1867	7320-37-8	57	9.78E+12	6318	3.33	0	4.14E-14
40	Eugenol	940	6054	97-53-0	164	9.52E+12	3156	3.00	0	0
41	Cogeijerene	908	2453	5988-83-0	147	8.88E+12	4224	3.36	0	0
42	Citronellyl isohexanoate	933	4070	71662-18-5	81	7.09E+12	4200	2.72	0	3.38E-14
43	Batilol	738	1199	544-62-7	57	6.76E+12	6414	3.20	0	1.44E-14
44	Neral	917	7158	106-26-3	69	6.40E+12	2472	2.62	0	0
45	α-epi-Muurolol	909	6241	19912-62-0	95	6.01E+12	4182	3.24	0	4.26E-14
46	Caryophyllene	959	5470	13877-93-5	93	5.98E+12	2862	3.01	0	3.05E-14
47	Citronellyl acetate	923	4231	150-84-5	81	5.54E+12	2754	2.60	0	1.77E-14
48	β-Farnesene	937	7187	18794-84-8	93	5.32E+12	3060	2.72	0	3.38E-14

49	Citronellyl butyrate	968	6181	141-16-2	81	4.94E+12	3528	2.78	0	3.31E-14
50	γ-Eudesmol	916	5594	1209-71-8	189	4.24E+12	4152	3.28	0	2.80E-14
51	Guaia-6,9-diene	943	2942	37839-64-8	105	4.07E+12	2922	3.11	0	0
52	Lavandulyl 2-methyl butanoate	919	3425	17609-96-0	69	4.05E+12	3846	2.91	0	3.16E-14
53	10-Nonadecanone	787	6043	504-57-4	57	3.95E+12	5028	2.65	0	1.73E-14
54	Junenol	913	5805	472-07-1	109	3.62E+12	3984	3.37	0	2.73E-14
55	Spathulenol	943	5262	72203-24-8	43	3.57E+12	3942	3.15	0	2.92E-14
56	Norbourbonone	898	9361	13844-03-6	81	3.25E+12	3882	3.41	0	2.70E-14
57	Geranic acid	958	7900	4698-08-2	69	3.01E+12	3570	1.94	0	2.37E-14
58	α-Agarofuran	967	9209	5956-12-7	82	2.99E+12	3576	3.69	0	2.49E-14
59	Epicubenol	927	5901	19912-67-5	161	2.71E+12	3972	3.25	0	0
60	δ-Cadinol	916	3122	19435-97-3	119	2.68E+12	4206	3.26	0	4.23E-14
61	p-Mentha-1,5-dien-8-ol	896	8805	1686-20-0	59	2.51E+12	2388	2.40	0	1.92E-14
62	Cubeban-11-ol	904	6334	220766-71-2	107	2.21E+12	3924	3.14	0	0
63	γ-Muurolene	888	2179	523-47-7	105	2.12E+12	3084	3.17	0	2.90E-14
64	1-Eicosanol	896	2292	629-96-9	57	2.09E+12	5478	2.83	0	3.25E-14
65	Caryophyllene oxide	887	5764	1139-30-6	79	2.04E+12	3906	3.30	0	2.79E-14
66	Ledene	882	3925	552-02-3	81	1.86E+12	3954	3.14	0	0
67	Methyl geranate	867	5936	1189-09-9	69	1.77E+12	2670	2.80	0	1.64E-14
68	10-epi-Elemol	918	4842	639-99-6	93	1.65E+12	3822	2.84	0	3.24E-14
69	α-Copaen-11-ol	838	8219	41370-56-3	59	1.63E+12	4392	3.36	0	0
70	7α-Hydroperoxy manool	638	1051	24583-54-8	69	1.56E+12	4896	3.08	0	2.98E-14
71	Menthoglycol	903	7860	92471-23-3	81	1.38E+12	3342	2.33	0	3.95E-14
72	Phenylethyl octanoate	907	8304	5457-70-5	104	1.35E+12	5022	3.49	0	3.95E-14
73	Isopulegol	822	4183	89-79-2	67	1.32E+12	2712	2.19	0	2.10E-14
74	β-Helmiscapene	936	1345	17627-30-4	133	1.25E+12	3126	3.34	0	2.75E-14
75	α-Copaene	919	4866	138874-68-7	93	1.22E+12	2568	2.84	0	3.24E-14
76	α-Calacorene	922	8869	21391-99-1	157	1.22E+12	3552	3.65	0	2.52E-14
77	*n*-Dodecenylsuccinic anhydride	635	2314	19780-11-1	57	1.15E+12	5124	2.62	0	0
78	Rose oxide II	891	9568	16409-43-1	139	1.14E+12	1656	2.39	0	0

79	neo-Intermedeol	942	4507	5945-72-2	81	1.13E+12	4296	3.33	0	4.14E-14
80	α-Agarofuran	866	8626	5956-12-7	82	1.06E+12	3264	3.56	0	2.58E-14
81	2-Phenylethyl (Z)-2-methyl-2-butenoate	903	2178	/	104	1.05E+12	4032	4.61	0	0
82	Cadina-1,4-diene	903	1798	38758-02-0	105	1.00E+12	3426	3.28	0	2.80E-14
83	Phytol acetate	807	1632	10236-16-5	95	9.83E+11	5958	3.12	0	1.47E-14
84	γ-Muurolene	895	1693	30021-74-0	93	8.98E+11	3318	3.28	0	2.80E-14
85	Spathulenol	797	3179	77171-55-2	105	8.96E+11	3732	3.56	0	2.58E-14
86	Isoledene	868	921	95910-36-4	105	8.84E+11	2958	3.16	0	2.91E-14
87	Lavandulyl 2-methyl butanoate	901	2186	17609-96-0	69	8.76E+11	4116	2.98	0	3.09E-14
88	Drim-8(12)-ene	830	5656	168113-16-4	95	8.69E+11	6336	4.57	0	2.01E-14
89	Octadecanal	871	1894	638-66-4	71	8.16E+11	5604	2.83	0	3.25E-14
90	Myrtenyl angelate	880	3777	138530-45-7	91	7.87E+11	4128	3.44	0	1.34E-14
91	Phellandral	889	6551	21391-98-0	81	7.87E+11	2706	2.75	0	0
92	*n*-Dodecanoic acid	722	3939	143-07-7	73	7.85E+11	6672	1.47	0	1.56E-14
93	*n*-Tridecanal	887	2101	10486-19-8	57	7.42E+11	4446	2.59	0	3.55E-14
94	Menthol	911	2818	1490-04-6	71	7.39E+11	2172	2.26	0	4.07E-14
95	Elemene	895	3272	515-13-9	81	7.37E+11	2730	2.77	0	3.32E-14
96	Terpinen-4-ol	910	6911	562-74-3	71	7.31E+11	2136	2.34	0	1.96E-14
97	14-Methylhexadec-8-enal	790	1437	60609-53-2	81	7.21E+11	5904	3.15	0	2.92E-14
98	γ-Elemene	812	1551	29873-99-2	93	6.95E+11	4428	3.59	0	5.12E-14
99	Viridiflorol	887	3886	19078-39-8	109	6.88E+11	3780	3.24	0	4.26E-14
100	Fokienol	888	2409	33440-00-5	55	6.84E+11	4764	3.01	0	3.05E-14

Table S3 Enantiomeric composition and separation factors of selected chiral terpenes in studied essential oils obtained with column set-up 2

Feature no.	Compound	*EC*, %					*k*
		AK3	IREMK1	IREMK2	IREMK4	IREMK5	
1	Citronellol I	71,88	74,59	72,83	75,67	66,75	1,04
2	Citronellol II	28,12	25,41	27,17	24,33	33,25	
3	Drim-8(12)-en e I	31,40	43,76	26,53	26,85	70,05	1,12
4	Drim-8(12)-ene II	68,60	56,24	73,47	73,15	29,95	

5	Guaia-6,9-diene I	29,10	51,27	44,56	60,71	46,03	1,01
6	Guaia-6,9-diene II	70,90	48,73	55,44	39,29	53,97	
7	Isomenthone I	61,98	63,15	20,16	56,96	53,59	1,08
8	Isomenthone II	38,02	36,85	79,84	43,04	46,41	
9	Rose oxide I	55,81	48,90	41,53	35,17	41,69	1,03
10	Rose oxide II	1,16	1,06	1,50	1,25	36,55	1,07
11	Rose oxide III	43,03	50,05	56,97	63,58	21,76	
13	Spathulenol II	72,60	92,57	6,03	55,60	27,64	1,89
12	Spathulenol I	27,40	7,43	93,97	44,40	72,36	
14	α-Terpineol I	44,16	48,06	41,15	46,27	33,65	1,02
15	α-Terpineol II	55,84	51,94	58,85	53,73	66,35	
16	β-Bourbonene I	8,52	37,68	20,69	23,27	16,93	1,01
17	β-Bourbonene II	90,42	51,59	41,51	48,45	43,98	1,40
18	β-Bourbonene III	1,06	10,73	37,80	28,28	39,09	
19	β-Eudesmol I	57,90	74,30	63,69	73,63	53,44	1,00
20	β-Eudesmol II	42,10	25,70	36,31	26,37	46,56	

Table S4 Enantiomeric composition and separation factors of selected chiral terpenes in studied essential oils obtained with column set-up 3

Feature no.	Compound	*EC*, %					*k*
		AK3	IREMK1	IREMK2	IREMK4	IREMK5	
1	Citronellyl formate I	42,60	30,42	30,93	36,83	53,47	1,0
2	Citronellyl formate II	57,40	69,58	69,07	63,17	46,53	
3	Linalool I	32,88	45,39	29,51	34,21	47,94	1,0
4	Linalool II	67,12	54,61	70,49	65,79	52,06	
5	Rose oxide I	65,07	66,93	64,28	63,47	76,12	1,1
6	Rose oxide II	34,93	33,07	35,72	36,53	23,88	
7	β-Eudesmol I	69,11	59,93	74,99	54,98	64,61	1,0
8	β-Eudesmol II	30,89	40,07	25,01	45,02	35,39	

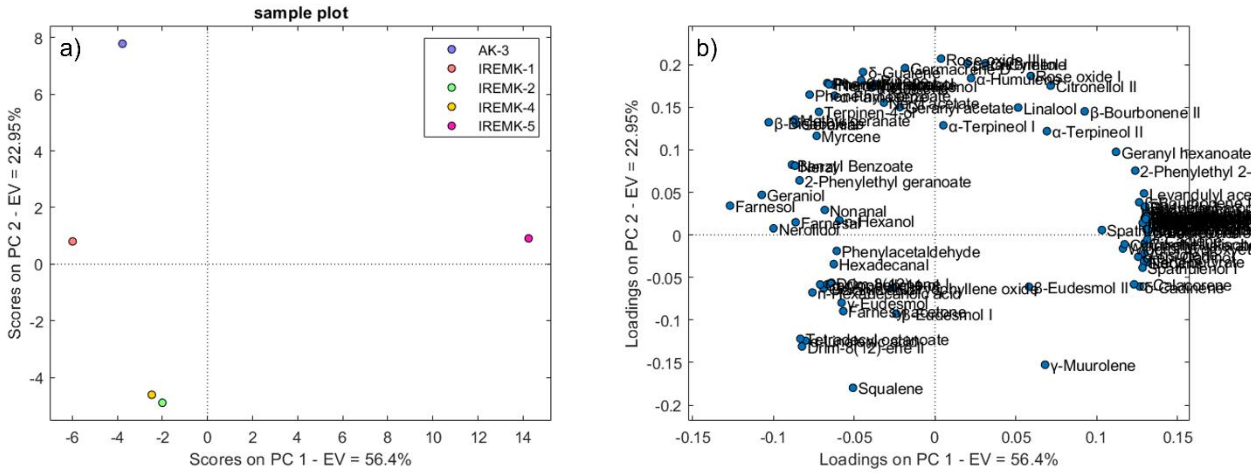

Figure S1 PCA results - scores a) and loadings b) plots for 100 identified features with indication of discrimination between different essential oils on column set-up 2

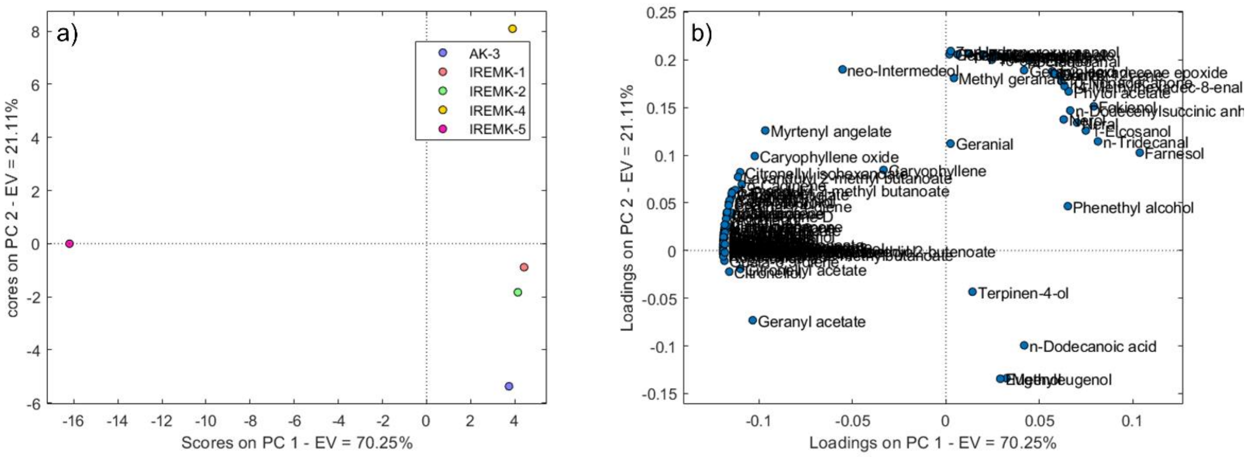

Figure S2 PCA results - scores a) and loadings b) plots for 100 identified features with indication of discrimination between different essential oils on column set-up 3

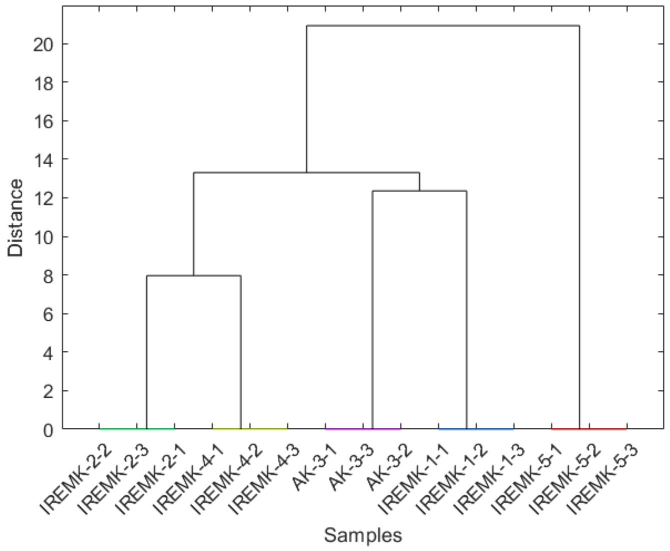

Figure S3 Dendrogram obtained for the reduced feature set obtained on column set-up 2

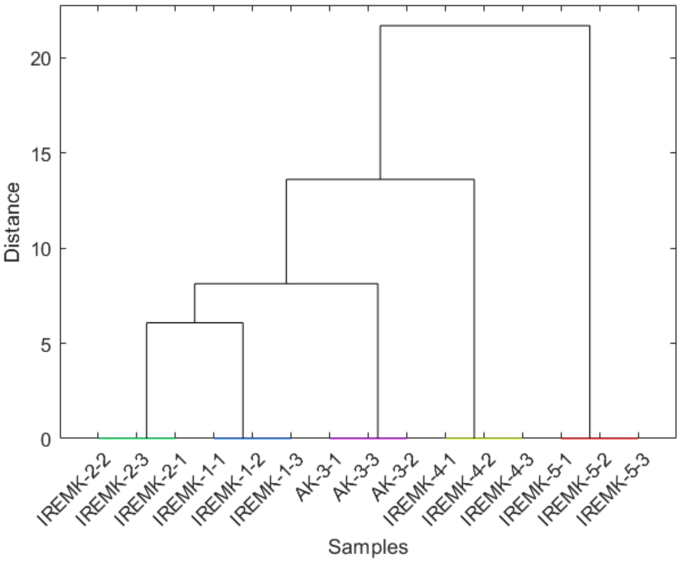

Figure S4 Dendrogram obtained for the reduced feature set obtained on column set-up 3

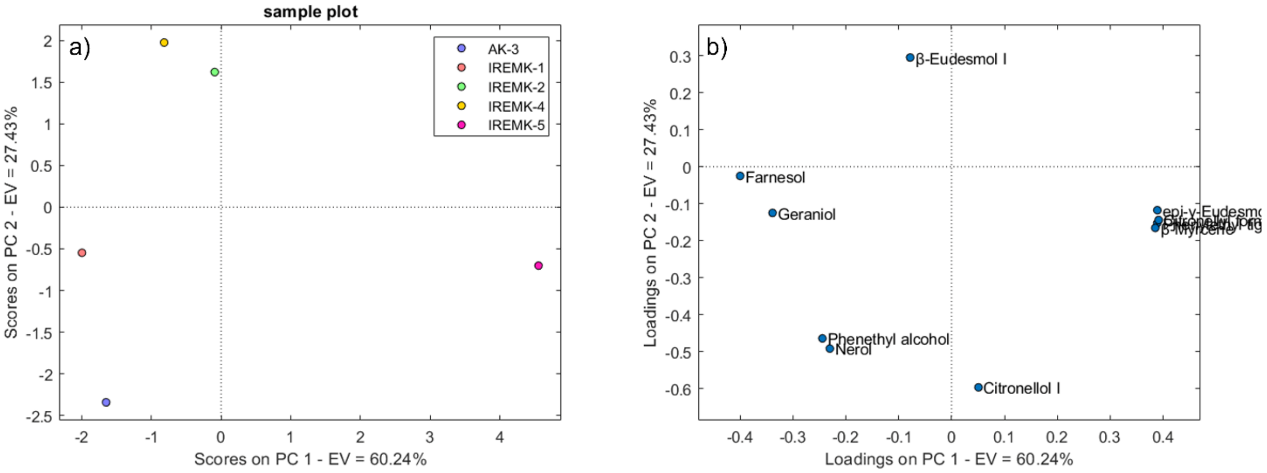

Figure S5 PCA results - scores a) and loadings b) plots for 10 identified features with indication of discrimination between different essential oils on column set-up 2

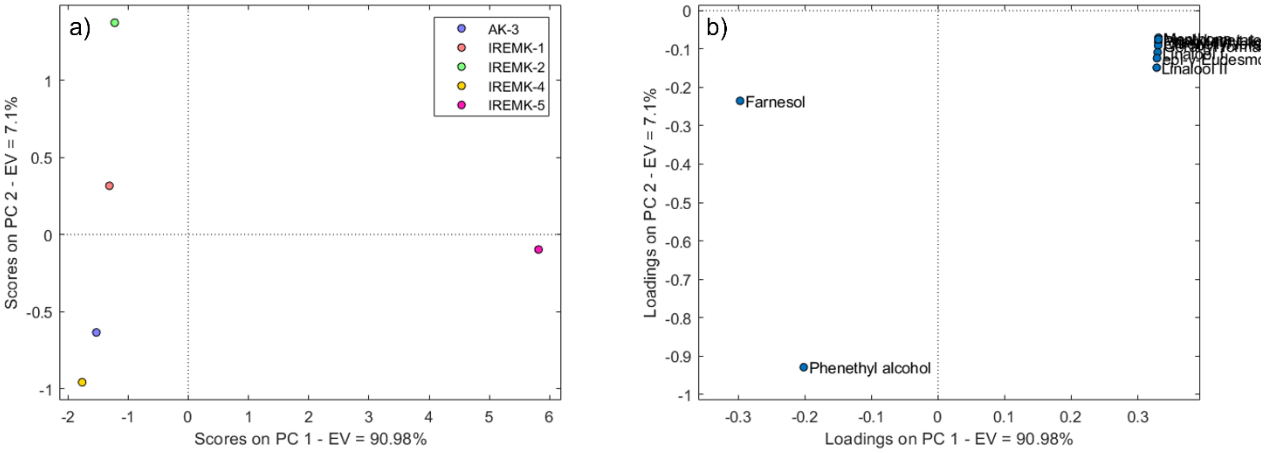

Figure S6 PCA results - scores a) and loadings b) plots for 10 identified features with indication of discrimination between different essential oils on column set-up 3

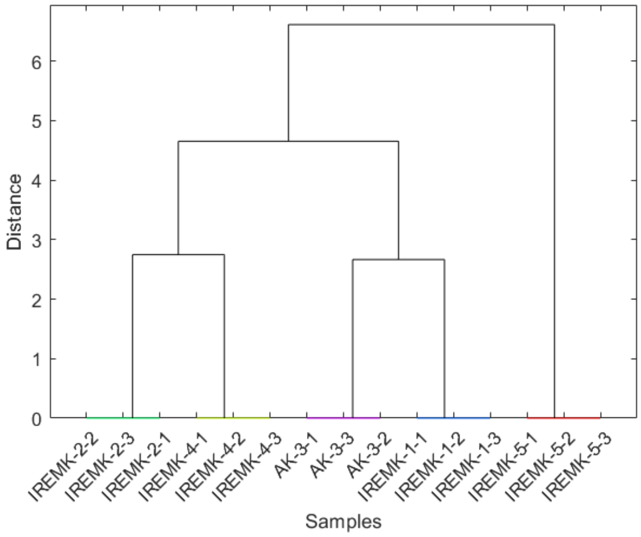

Figure S7 Dendrogram obtained for the 100-feature set obtained on column set-up 2

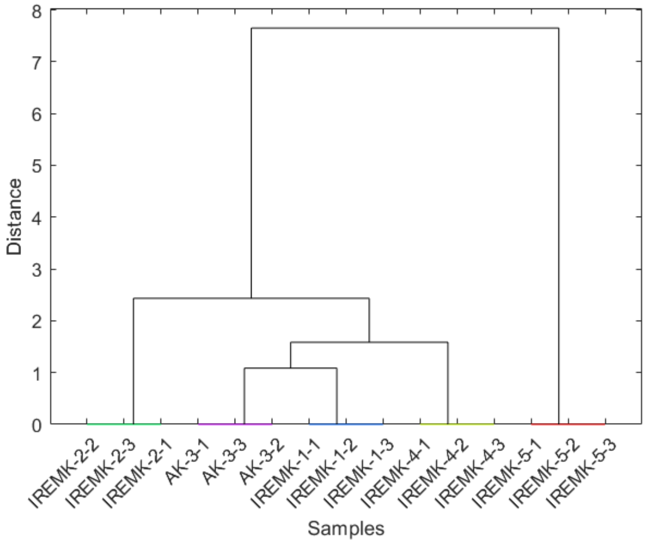

Figure S8 Dendrogram obtained for the 100-feature set obtained on column set-up 3
